# Beneficial Effect of *Thymelaea hirsuta* on Pancreatic Islet Degeneration, Renal Fibrosis, and Liver Damages as Demonstrated in Streptozotocin-Induced Diabetic Rat

**DOI:** 10.1155/2021/6614903

**Published:** 2021-02-18

**Authors:** Sanae Abid, Hassane Mekhfi, Abderrahim Ziyyat, Abdekhaleq Legssyer, Mohammed Aziz, Mohamed Bnouham

**Affiliations:** Laboratory of Bioresources, Biotechnology, Ethnopharmacology and Health, Department of Biology, Faculty of Sciences, University Mohamed Ist, Bd: Mohamed VI, BP: 717, Oujda 60000, Morocco

## Abstract

**Objective:**

In Morocco, *Thymelaea hirsuta* (*T. hirsuta*) (Thymelaeacea) is a medicinal plant widely used to treat and prevent diabetes. The present study aimed to evaluate the medium-term antidiabetic effect of aqueous extract (AqTh) and ethyl acetate fraction (EaTh) of Th and to investigate their putative protective effect on pancreatic islet degeneration, diabetic nephropathy, and liver damages in streptozotocin (STZ)-diabetic rats.

**Methods:**

Experimental diabetes in rats was induced by a single intraperitoneal injection of 50 mg/kg of STZ. During the treatment period (4 weeks), 200 mg/kg AqTh and 50 mg/kg EaTh were orally administrated daily to STZ-diabetic rats. A group of parameters including fasting blood glucose, biochemical parameters, and intestinal *α*-glucosidase inhibition were studied. Furthermore, histological study of the pancreas, kidney, liver, and aorta was also realized.

**Results:**

At the end of the treatment, both AqTh and EaTh had normalized fasting blood glucose to 1.08 and 1.25 g/l, respectively. AqTh has also reduced urinary creatinine and HbAc1. The EaTh showed inhibitory activity against intestinal *α*-glucosidase, whereas AqTh did not have this inhibitory effect. Furthermore, pancreas hematoxylin and eosin staining showed that AqTh or EaTh prevents pancreatic islet cell degeneration. As the same kidney, Masson's trichrome staining has shown a significant prevention of renal fibrosis in AqTh- or EaTh-treated diabetic rats. On the other hand, liver hematoxylin and eosin staining showed that AqTh and EaTh prevent liver damage.

**Conclusion:**

We conclude that medium-term administration of AqTh and EaTh exerts significant antihyperglycemic effect in STZ-diabetic rats possibly through intestinal *α*-glucosidase inhibition and protection against pancreatic islet cell damage. Moreover, AqTh and EaTh treatment prevent nephropathy and liver complications in STZ-diabetic rats.

## 1. Introduction

Diabetes mellitus is the most common endocrine disease. The incidence of this disease is increasing at an alarming rate (4-5%) [1]. Untreated or noncontrolled, noninsulin-dependent type 2 diabetes (DM2) can cause several complications such as cardiovascular diseases and diabetic nephropathy, and it may lead to insulin-dependent type 1 diabetes (DM1).

The diabetic nephropathy is one of the important complications of diabetes, and it is the main reason for the increased number of patients with end-stage renal disease (ESRD) [2], which require kidney dialysis or a kidney transplant for the patient to live.

Therefore, the management of diabetes is becoming very important to avoid its serious metabolic outcomes such as nephropathy.

Nowadays, the treatment of diabetes involves diet control, exercise, the use of insulin and/or hypoglycemic drugs, and also the use of medicinal plants as complementary alternative medicine. Several medicinal plants have shown a crucial antihyperglycemic effect with minimal side effects [3–5], For this reason, the World Health Organization (WHO) has called a great attention to the rational use of traditional and natural medicines for treating diabetes [6].

In Morocco, more than 100 medicinal plants are used to treat and prevent diabetes [7–10]. The antihyperglycemic and antidiabetic activity of numerous extracts and products of these plants (*Ammi visnaga* Lam., *Globularia alypum*, *Nigella sativa*, and *Olea europaea* var.) has been confirmed [9].

*T. hirsuta* is a medicinal plant traditionally used for the prevention and the management of diabetes, in north-eastern Morocco [8]. Previous pharmacological studies have demonstrated the acute antihyperglycemic effect of the AqTh and EaTh [11–13]. However, the medium-term antidiabetic effect of these two *T. hirsuta* extracts has not yet been established. Therefore, the aim of the present study is to evaluate for the first time the medium-term antidiabetic activity of the AqTh and EaTh and to investigate their putative protective effect on pancreatic islet degeneration, diabetic nephropathy, liver steatosis, and aorta complications in STZ-induced diabetic rats.

## 2. Materials and Methods

### 2.1. Chemicals and Reagents

Glucose Autokit was purchased from BioSystems (Spain). STZ was purchased from Sigma-Aldrich (China). Acarbose (Glucor 50) was obtained from Bayer Schering Pharma (Casablanca, Morocco). Sucrose was purchased from Prolabo (groupe Rhone-Poulenc) (EEC). Pentobarbital was obtained from CEVA santé animale (La Ballastière). Anthrone (C14H10O) was purchased from Acros Organics. Paraffin wax was purchased from Fluka Chemika (Switzerland). Eosin (C_20_H_6_Br_4_Na_2_O_5_) was obtained from Riedel-de Haen (Seelze), and hematoxylin was obtained from BDH Chemicals Ltd. (Poole England). Fuschsin acid was purchased from Acros Organics (New Jersey, USA), phosphomolybdic acid was purchased from Sigma-Aldrich (PF, Steinheim), and light green was purchased from Sigma-Aldrich (MO, USA).

### 2.2. Animals

Wistar rats of both genders, initially weighing 150–250 g (8-9 weeks), were obtained from the animal house of the Department of Biology of the Faculty of Sciences (Oujda, Morocco). The study has been carried out along the “Principles of Laboratory Animal Care” [14].They were maintained under standard laboratory conditions (light/dark cycle of 12/12 h and temperature of 23 + 2°C with 3 rats per cage with access to food and water. To collect urine and determine water and food intake, rats were maintained in metabolic cages.

### 2.3. Induction of Diabetes

After a 14 h fasting, the rats were intraperitoneally injected with a single dose of STZ (50 mg/kg bw), prepared in a fresh and cold sodium citrate buffer (0.1 M citric acid and 0.1 M trisoduim citrate dihydrate) at pH 4.5, to induce diabetes [15]. After 1 week, rats with fasting blood glucose range of 1.26–2 g/l were considered as type 2 diabetic and they were included in the study.

### 2.4. Preparation of Plant Sample

*T. hirsuta* was purchased from a traditional market in Oujda (Oriental Morocco) and was authenticated by a botanist in the Department of Biology (Faculty of Sciences, Oujda, Morocco), and a voucher specimen (HUMPOM137) was deposit at the plant section of the Herbarium University Mohamed Premier of Oujda, Morocco (HUMPO).

*T. hirsuta*'s aerial parts were first cleaned and washed with water and then dried at 40°C overnight in the oven. To prepare AqTh, 140 g of *T. hirsuta* aerial parts was infused in 2 L of distillated water for 3 hours. The AqTh yield was 4.53%. The EaTh was prepared as described in our previous study [13].

### 2.5. Experimental Design

The rats were randomly divided into five groups (5 or 6 rats per group): normal control rats (administered only with distillated water), diabetic control rats (administered only with distillated water), diabetic rats treated with 10 mg/kg bw of acarbose (standard hypoglycemic drug), diabetic rats treated with 50 mg/kg of EaTh, and diabetic rats treated with 200 mg/kg bw of AqTh. Optimal doses were determined based on our previous studies [13] and from preliminary tests. All rats were treated once daily for 4 weeks. The body weight was recorded every day. The water and food intake and urine volume were monitored before and after treatment, and glycosuria was measured at the end of the treatment. Fasting blood glucose was examined before the treatment (week 0) and after 1, 2, 3, and 4 weeks of treatment. The inhibition of *α*-glucosidase, *in vivo*, was studied the day before rats were sacrificed. After the 4 weeks of treatment, all the animals were anesthetized by 50 mg/kg of pentobarbital, after 14 h of fasting. Then, the blood was collected, by cardiac puncture, and immediately centrifuged at 3000 tour/min for 10 min. The serum was then stored at −20°C until biochemical analysis (total cholesterol and triglycerides). The kidney, liver, aorta, heart, and pancreas were removed, weighted (kidney liver and heart), and then fixed (kidney, liver, aorta, and pancreas) in 10% formalin for the histological study. A liver's sample was stored at −20°C for glycogen content's assay.

### 2.6. Estimation of Fasting Blood Glucose

During the treatment period, the fasting glycemia was quantified weekly. After 14 h of fasting, blood was removed from the tail vein rats, under light ether anesthesia, using microcapillaries. Then, it was centrifuged at 5000 ×g for 10 min, and glycemia was estimated in the serum using a commercially glucose kit (BioSystems, Spain) based on the glucose oxidase peroxidase method [16].

### 2.7. *α*-Glucosidase Inhibition, *in vivo* Study

The day before the sacrifice, the rats deprived of food for 14 h were used to monitor the *α*-glucosidase inhibition, *in vivo*. 30 min prior oral sucrose load (2 g/kg), each group was administrated by the dose corresponding to its treatment. The blood was collected, from the tail vein using microcapillaries, just before the test dose administration (−30 min), immediately before sucrose load (0 min), and at 30, 60, and 120 min after sucrose load. After centrifugation at 5000 ×g/10 min, the plasma glucose level was determined by the glucose oxidase peroxidase method [17].

### 2.8. Extraction and Determination of Hepatic Glycogen

Weighed samples of liver tissues (0.3–0.5 g) from all groups were used to extract glycogen according to Ong and Khoo [18]. Liver samples were first crushed, homogenized with 2 ml of 30% potassium hydroxide (KOH), and boiled at 100°C/30 min. Then, for precipitating glycogen, the mixture was treated twice with 4 ml 95% ethanol, and each time, the mixture was stored at 4°C/30 min. After centrifugation at 3000 tours/min/15 min, the pellet was washed with 8 ml 95% ethanol and then centrifuged at 3000 tours/min/15 min. The glycogen obtained was solubilized in 1 ml distillated water. The glycogen concentration was monitored using anthrone reagent. The optic density was read at 625 nm.

### 2.9. Biochemical Assays

Glycosuria was measured by a commercial Autokit (BioSystems, Spain) based on the glucose oxidase peroxidase method. Glycosylated hemoglobin (HbA1c) was estimated using a commercial kit (Cal-tech Diagnostics, INC, USA), and cholesterol was determined using commercial assay kits (SGM- Italia, Roma, Italy). Triglycerides and urinary creatinine were estimated using commercial assays kits (Bio Sud Diagnostici S.R.L, Ricerca, Italy).

### 2.10. Histopathology

After 3 ± 1 day's period fixation in 10% formalin, the pancreas, kidney, liver, and aorta tissues were washed by distillated water for 20 min. Then, they were dehydrated with increasing ethanol title (30% for 30 min, 70% for 30 min, 95% for 30 min, and 2 × 100% for 60 min, respectively). After an enlightenment step in toluene (2 × 120 min), organs were included by the mixture paraffin-toluene (1V/1V) for 90 min, then by paraffin (2 × 120 min). Finally, the organs were embedded in paraffin before sectioning at 7 *μ*m (microtome Leitz 1512). Pancreas and liver sections were stained with hematoxylin and eosin. Kidney and aorta sections were stained with Masson's trichrome to show fibrosis/collagen.

Before staining with hematoxylin and eosin, the sections were deparaffinized and incubated in toluene (2 × 5 min), then hydrated by decreasing ethanol title (100%, 95%, and 70%, respectively) for 5 min. Then, for hematoxylin and eosin staining, after 20 min of washing, cell nuclei were stained with immersed in hematoxylin for 5 min, followed by 15 min of washing by water. To stain cytoplasm, the sections were immersed in 1% eosin for 5 min, then washed for 2 min. After coloration, sections were dehydrated with 100% ethanol (2 × 1/2 min) and toluene (2 × 2 min). For Masson's trichrome staining, after the hydratation, kidney and aorta sections were refixed in Bouin's solution for 1 hour/56°C. Then, after 10 min washing, sections were stained in Weigert's iron hematoxylin for 2 min. After 10 min rinsing, sections were immersed in fuchsin acid for 3 min, and after 10 min washing, they were differentiated in phosphomolybdic acid for 15 min. To stain collagen, sections were transferred directly to light green for 15 min. After 2 min differentiation in 1% acetic acid, sections were dehydrated through 95% and 100% ethanol for 1 min each and then cleared in toluene (2 × 1 min) [19–21].

Finally, sections stained on lamellas were mounted in Permount. After drying, the microscopic observation was made using the 40x objective ocular system of Olympus Tokyo (Japan) light microscope.

To determine drug's effect on pancreas tissue, the pancreatic islet cell number and diameter were calculated. To quantify collagen on kidney sections, results were scored from zero to three (0: no collagen, 1: weak collagen, 2: moderate collagen, and 3: strong collagen) [22]. The treatment effect on aorta was evaluated by measuring the aorta diameter, and liver damage was elucidated by the development of lipid droplets.

### 2.11. Statistical Analysis

All the values were expressed as mean ± SEM. The statistical analysis and comparison of means was performed using the unpaired Student test to compare two groups' means and paired Student test to compare two means inside the same group. To determine the protective drug effect on the kidneys and liver, the chi-square test was used. *p* values <0.05 were considered significant.

## 3. Results

### 3.1. Effect of Drug Treatment on Fasting Blood Glucose in STZ-Induced Diabetic Rats

**Figure 1:**
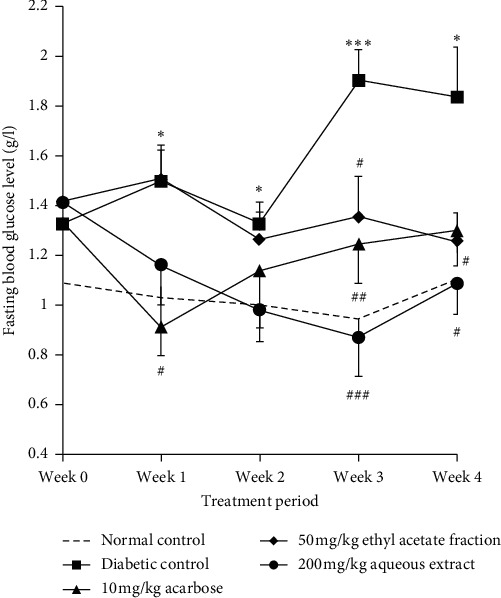
Effect of daily oral administration of aqueous extract (AqTh), ethyl acetate fraction (EaTh) of *T. hirsute*, and acarbose during 4 weeks on fasting blood glucose in STZ-diabetic rats. Rats were deprived of food for 14 h, and then blood samples were collected from the tail tip under light ether anesthesia. Each value represents the means ± SEM. *n* = 5-6. ^*∗*^*p* < 0.05 and ^*∗∗∗*^*p* < 0.001 compared with the normal group at each point. ^#^*p* < 0.05, ^##^*p* < 0.01, and ^###^*p* < 0.001 compared with the diabetic group at each point.

In untreated diabetic control rats, a significant fasting blood glucose elevation was observed compared to normal control rats. Treatment with 200 mg/kg AqTh decreased blood glucose level from the 1^st^ week of administration, and this effect was significant at the 3^rd^ (*p* < 0.001) and 4^th^ week (*p* < 0.05) compared to untreated diabetic rats. Treatment with 50 mg/kg EaTh reduced hyperglycemia from the 2^nd^ week, and this effect was significant (*p* < 0.05) at the 3^rd^ and 4^th^ week. At 2^nd^, 3^rd^, and 4^th^ week, EaTh effect was statistically similar to that of 10 mg/kg acarbose (Figure 1).

### 3.2. Effect of Drugs on *α*-Glucosidase Activity, *in vivo* Study

**Figure 2:**
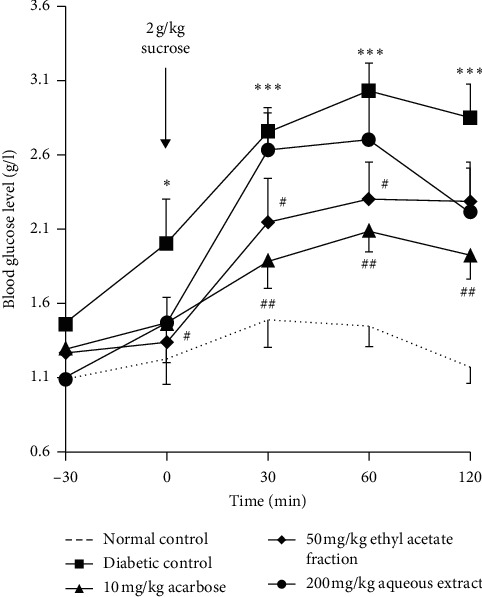
Effect of aqueous extract (AqTh), ethyl acetate fraction (EaTh) of *T. hirsute*, and acarbose on blood glucose level after sucrose loading in STZ-induced diabetic rats. Each value represents the means ± SEM. *n* = 5-6. ^*∗*^*p* < 0.05 and ^*∗∗∗*^*p* < 0.001 compared with the normal group at each point. ^#^*p* < 0.05 and ^##^*p* < 0.01 compared with the diabetic group at each point. All statistical tests were used with one-tail *p* value.

In normal control rats, blood glucose was increased to 1.48 g/l.30 min after sucrose loading and then it returns to normal value at 120 min whereas in untreated diabetic rats, glycemia was increased to 2.76 g/l at 30 min of sucrose loading to achieve 2.85 g/l at 120 min. 50 mg/kg EaTh has significantly (*p* < 0.05) prevented the hyperglycemia induced by sucrose loading at 30 and 60 min compared with untreated diabetic rats. In the 200 mg/kg AqTh group, no antihyperglycemic effect was shown. Blood glucose level reached 2.63 g/l and 2.70 g/l at 30 and 60 min, respectively, and it decreased to 2.21 g/l at 120 min. 10 mg/kg acarbose has significantly (*p* < 0.01) prevented the hyperglycemia induced by sucrose loading at 30, 60, and 120 min compared to diabetic control. The effect of acarbose was statistically similar to EaTh effect (Figure 2).

### 3.3. Effect of Drug Treatment on Body Weight, Food, and Water Intake

The body weight gain by untreated diabetic rats was significantly (*p* < 0.05) decreased compared with normal rats. AqTh has significantly (*p* < 0.05) prevented the body weight fall compared with untreated diabetic rats. EaTh- and acarbose-treated rats did not significantly (*p* > 0.05) change the body weight gain compared to untreated diabetic rats. At the end of treatment, water intake did not change in untreated diabetic rats, AqTh, EaTh, and acarbose groups compared to the data before treatment whereas food intake was significantly increased in diabetic rats and AqTh-, EaTh-, and acarbose-treated rats (*p* < 0.05, *p* < 0.05, *p* < 0.05, and *p* < 0.01, respectively) compared to before drug administration (Table 1).

### 3.4. Effect of Drug Treatment on Urinary Parameters

After the 4-week treatment period, urinary creatinine and glycosuria were significantly increased (*p* < 0.05 and *p* < 0.001, respectively) in untreated diabetic rats when compared to normal rats. Oral administration of AqTh has decreased significantly (*p* < 0.05) urinary creatinine compared to untreated diabetic rats, but no significant difference has observed on glycosuria when compared with untreated diabetic rats. EaTh treatment has prevented urinary creatinine increase compared to normal rats; however, EaTh did not change significantly glycosuria. On the other hand, the AqTh and EaTh effect on urinary creatinine and glycosuria was statistically similar to that of 10 mg/kg acarbose. Finally, there was no significant difference on urinary volume when comparing data before and after treatment with AqTh, EaTh, or acarbose (Table 2).

### 3.5. Effect of Drug Treatment on Biochemical Parameters

After the 4-week treatment period, HbA1c was significantly increased (*p* < 0.05), in untreated diabetic rats when compared to normal rats. Oral administration of AqTh has prevented HbA1c elevation compared to normal rats. At the end of the treatment period, liver glycogen quantity was significantly decreased (*p* < 0.05) in untreated diabetic rats when compared to normal rats. AqTh and EaTh treatments have shown no significant effect on liver glycogen compared to untreated diabetic rats whereas acarbose treatment did not change liver glycogen quantity compared to normal rats. Finally, total cholesterol and triglycerides results show that there is no significant change after AqTh, EaTh, or acarbose treatment compared to normal and diabetic rats (Table 3).

### 3.6. Effect of Drug Treatment on Organ Weight

Table 4 shows liver, kidney, and heart weight at the end of the treatment period. Results indicate that kidney weight was significantly increased in untreated diabetic rats as compared to normal rats. Treatment with 200 mg/kg AqTh or 50 mg/kg EaTh did not change liver, kidney, and heart weight when compared to untreated diabetic rats whereas there is a significant elevation in liver and heart weight of acarbose-treated rats compared to untreated diabetic rats.

### 3.7. Histopathological Observation

#### 3.7.1. Pancreas Changes

**Figure 3:**
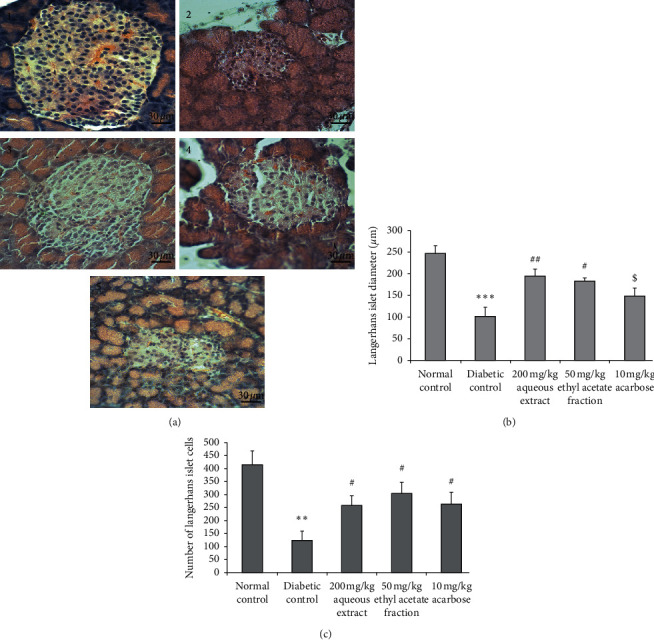
Effect of 200 mg/kg aqueous extract (AqTh), 50 mg/kg ethyl acetate (EaTh), and 10 mg/kg acarbose treatment on diameter and number of pancreatic Langerhans islet cells in STZ-induced diabetic rats after 4-week treatment. (a) Histological appearance of Langerhans islets in normal (1), diabetic (2), AqTh-treated (3), EaTh-treated (4), and acarbose-treated rats (5). (b) Diameter of Langerhans islets for all groups. (c) Number of Langerhans islet cells for all groups. Sections were stained in hematoxylin and eosin and observed in light microscope (original magnification ×400). ^*∗∗*^*p* < 0.01*and*^*∗∗∗*^*p* < 0.001 compared to normal rats; ^$^*p* > 0.05, ^#^*p* < 0.05, and ^##^*p* < 0.01 compared to diabetic rats. Each value represents the means ± SEM. *n* = 5-6.

Light microscopic observation of pancreatic islet cells from normal rats shows normal histological appearance with large diameter and high granulation density (Figure 3). In untreated diabetic rats, a significant reduction of diameter and islet cell number (*p* < 0.001 and *p* < 0.01, respectively) was observed. Treatments with 200 mg/kg AqTh have significantly prevented the reduction of diameter and cell number of Langerhans islet (*p* < 0.01 and *p* < 0.05, respectively). As the same, 50 mg/kg EaTh has significantly (*p* < 0.05) protected diameter and cell number against reduction. Treatment with 10 mg/kg acarbose has prevented significantly (*p* < 0.05) the reduction of the islet cell number.

#### 3.7.2. Kidney Changes

**Figure 4:**
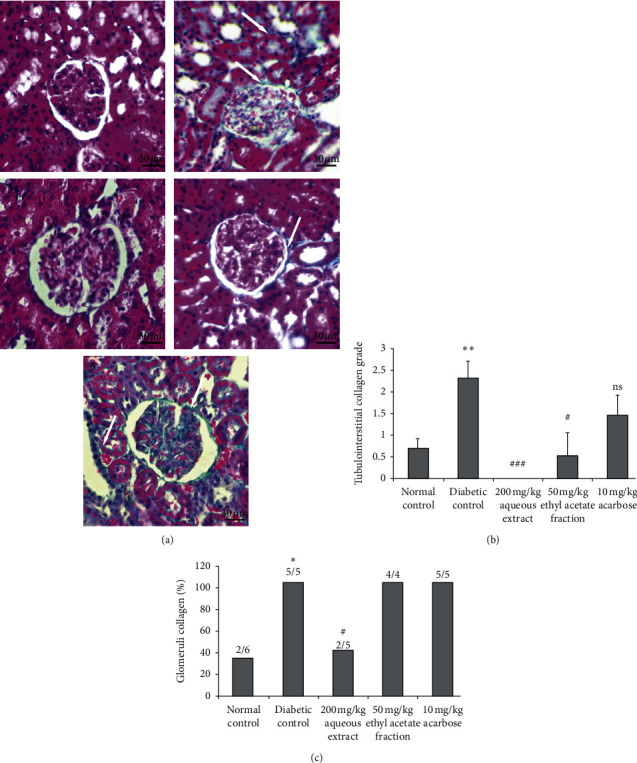
Effect of 200 mg/kg AqTh, 50 mg/kg EaTh, and 10 mg/kg acarbose treatment on glomeruli and tubulointerstilial collagen in the kidneys of STZ-induced diabetic rats after 4-week treatment. (a) Histological appearance of renal tubules and glomerules in normal (1), diabetic (2), AqTh-treated (3), EaTh-treated (4), and acarbose-treated rats (5). (b) Tubulointerstitial collagen grade for all groups. (c) Glomeruli collagen for all groups. Sections were stained in Masson's trichrome and observed in light microscope (original magnification ×400). White arrow: collagen. Each value represents the means ± SEM. *n* = 5-6. x/x: number of rats with glomeruli collagen in the group. ^*∗*^*p* < 0.05 and ^*∗∗*^*p* < 0.01 compared to normal rats. ^ns^*p* > 0.05 compared to diabetic rats; ^#^*p* < 0.05 and ^###^*p* < 0.001 compared to diabetic rats. All statistical tests were used with one-tail *p* value.

The Masson staining marks in blue/green collagen fibers, in brown nuclei, in red muscle fiber cytoplasm, and in orange blood cells. Minimal tubulointerstitial collagen was observed in the normal group (Figure 4). A significant increase (*p* < 0.01) of tubulointerstitial collagen was detected in untreated diabetic rats. Treatment with 200 mg/kg AqTh and 50 mg/kg EaTh has prevented significantly (*p* < 0.001 and *p* < 0.05, respectively) the tubulointerstitial collagen compared to untreated diabetic rats, but 10 mg/kg acarbose treatment did not change significantly (*p* > 0.05) this tubulointerstitial collagen. On the other hand, glomeruli collagen was observed in 33.3% of normal rats. In diabetic rats, glomeruli collagen was detected in all rats (100%). Treatment with 200 mg/kg AqTh has significantly reduced (*p* < 0.05) glomeruli collagen to 40%, but EaTh and acarbose did not (Figure 4).

#### 3.7.3. Liver Changes

**Figure 5:**
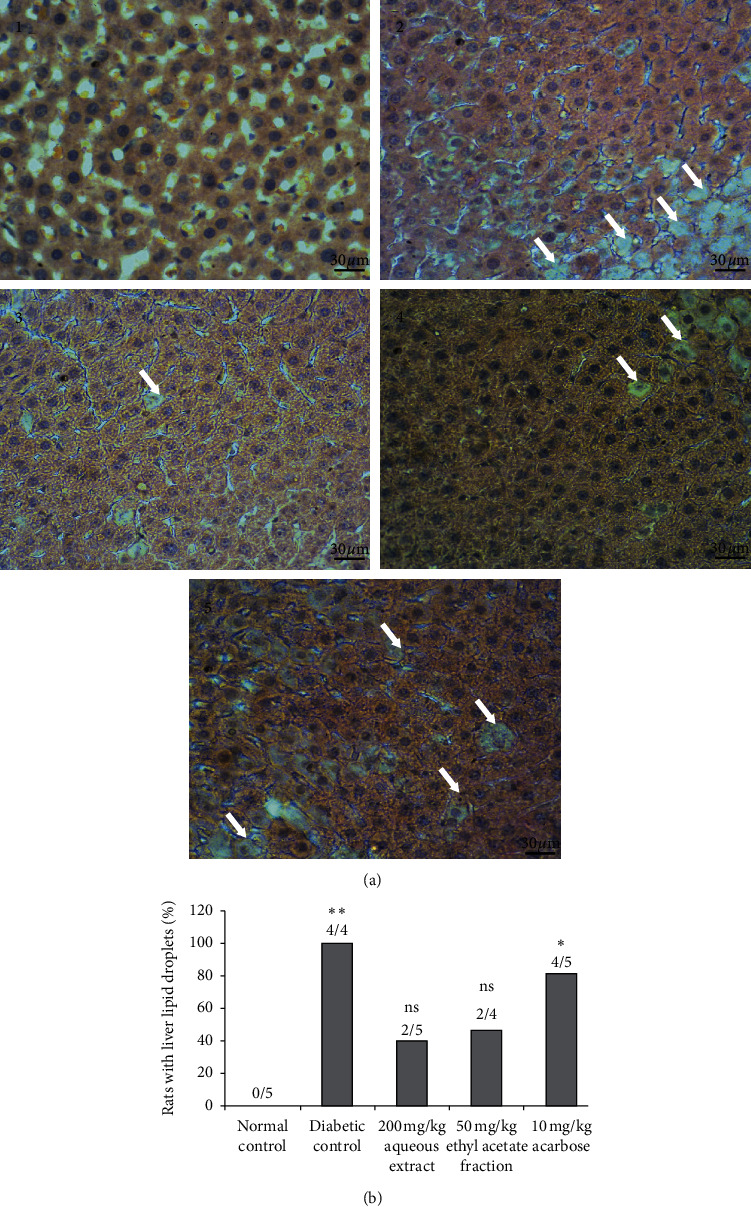
Effect of 200 mg/kg AqTh, 50 mg/kg EaTh, and 10 mg/kg acarbose treatment on the liver of STZ-induced diabetic rats after 4-week treatment. (a) Histological appearance of liver tissue in normal (1), diabetic (2), AqTh-treated (3), EaTh-treated (4), and acarbose-treated rats (5). (b) Percentage of rats with liver lipid droplets. Sections were stained in hematoxylin and eosin and observed in light microscope (original magnification ×400). White arrow: lipid droplets. Each value represents the means ± SEM. *n* = 4–6. x/x: number of rats with lipid droplets in the group. ^*∗*^*p* < 0.05 and ^*∗∗*^*p* < 0.01 compared to normal rats. ^ns^*p* > 0.05 compared to normal rats. All statistical tests were used with one-tail *p* value.

Examination of hematoxylin and eosin stained sections showed that normal rats had a normal histological appearance. In untreated diabetic rats, an important degenerative change in the liver tissue was observed. Lipid droplets were appeared in all the rats (100%). Treatment with 200 mg/kg AqTh and 50 mg/kg EaTh has significantly (*p* < 0.05) attenuated this effect. The percentage of protection was 60 and 50%, respectively. However, 10 mg/kg acarbose treatment did not attenuate this liver damage (Figure 5).

#### 3.7.4. Aorta Changes

**Figure 6:**
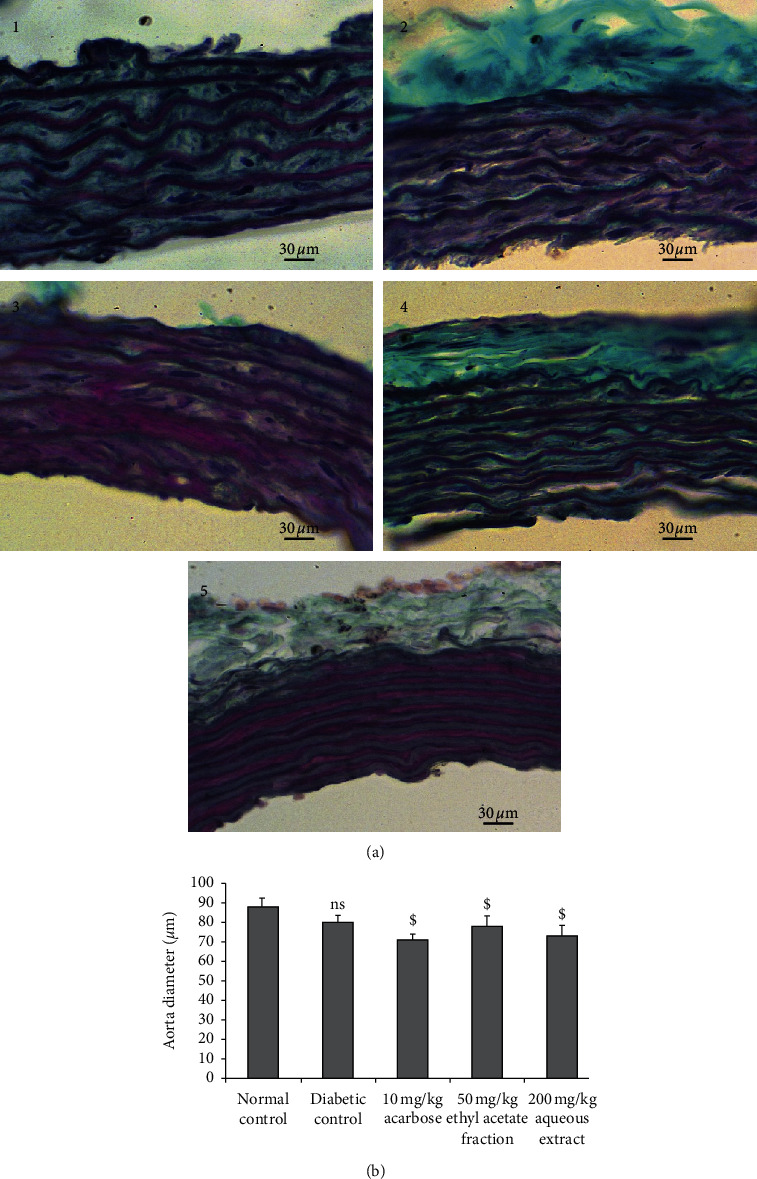
Effect of 200 mg/kg AqTh, 50 mg/kg EaTh, and 10 mg/kg acarbose treatment on aorta diameter of STZ-induced diabetic rats after 4-week treatment. (a) Histological appearance of aorta tissue in normal (1), diabetic (2), AqTh-treated (3), EaTh-treated (4), and acarbose-treated rats (5). (b) Aorta diameter. Sections were stained in Masson's trichrome and observed in light microscope (original magnification ×400). Each value represents the means ± SEM. *n* = 5-6. ^ns^*p* > 0.05 compared to the normal group. ^$^*p* > 0.05 compared to the diabetic group.

Light microscopic observation shows that untreated diabetic rats had normal aorta diameter compared to normal rats. Treatment with 200 mg/kg AqTh, 50 mg/kg EaTh, or 10 mg/kg acarbose did not significantly change aorta diameter compared to normal and diabetic rats (Figure 6).

## 4. Discussion

In this study, we demonstrated for the first time the antidiabetic effect of *T. hirsuta* medium-term administration (4 weeks) and its prevention of renal and liver complications in STZ-induced diabetic rats.

Our results indicated that the medium-term treatment with 200 mg/kg AqTh or 50 mg/kg EaTh decreases significantly fasting blood glucose in STZ-treated diabetic rats comparing with STZ-untreated diabetic rats. EaTh has shown a similar *α*-glucosidase inhibitory activity when compared to 10 mg/kg acarbose, but AqTh has not inhibited *α*-glucosidase activity after sucrose loading. These results indicate that the medium-term antihyperglycemic effect of EaTh is related to intestinal *α*-glucosidase inhibition. So, this finding confirms our previous results [13] whereas the antidiabetic effect of AqTh seems to be related to another mechanism than the *α*-glucosidase pathway. In addition, we have observed a reduction on HbAc1 level in AqTh-treated diabetic rats while EaTh treatment did not change HbAc1 level when compared to untreated diabetic rats. This explains why *α*-glucosidase inhibitors are often prescribed with other antidiabetic drugs in diabetic treatment.

Histopathological study of the sections of pancreas showed that the administration of AqTh and EaTh for 4 weeks significantly increases diameter and cell number in Langerhans islets. Similar studies had proved that many plant extracts significantly restored the diameter of islets and the number of *β* cells in diabetic rats [23–25].

Consequently, the plausible mechanism of action of AqTh in controlling the blood glucose level might be the enhancement of secretion of insulin from pancreatic *β*-cells. As other possible mechanisms, the AqTh may sensitize the insulin receptor to insulin or stimulate the stem cells of the islets of Langerhans in the pancreas of STZ-induced diabetic rats [25].

Therefore, we conclude that the antihyperglycemic phytochemical compounds present in AqTh are different to those of EaTh.

Many studies proved that the secondary metabolites such as flavonoids, tannins, and terpenoids have potential inhibitory effects on alpha glucosidase [26–28]. In a previous study of our team, a polyphenol‐rich fraction of *T. hirsuta* has demonstrated a potent antidiabetic effect in diabetic rats [29]. Thus, the *α*-glucosidase inhibitory action of EaTh is due probably to the presence of flavonoids.

It is well known that diabetes is associated with macrovascular and microvascular complications such as nephropathy. Statistics showed that of all dialysis patients, diabetics represent 20.6% in 2001 against 13.1% in 1995 and 6.9% in 1989. The mortality rate of patients on dialysis is significantly higher in diabetics than in nondiabetes (241.4/1000 vs. 153.99/1000 person-years) [30]. The average survival of type 2 diabetes entering dialysis is approximately 3 years [31, 32]. Diabetic nephropathy is characterized by the accumulation of some proteins such as collagen in the glomerular mesangium and in the tubulointerstitial space that lead to renal fibrosis and so to renal failure [33–35]. In this study, we have demonstrated that medium-term oral administration of 200 mg/kg AqTh or 50 mg/kg EaTh prevents urinary creatinine increasing and tubulointerstitial renal collagen when compared to untreated diabetic rats as shown in kidney histopathological results. These finding demonstrate that AqTh and EaTh could protect against renal fibrosis through the inhibition of the extracellular matrix (ECM) protein accumulation. These results are similar to other ethnopharmacological studies which demonstrated that garlic and *Sclerocarya birrea* ameliorate the process of renal fibrosis in diabetic nephropathy [36, 37]. The protective effect against renal ECM protein accumulation is due to the inhibition of the protein kinase B/mammalian target of the rapamycin (PKB/mTOR) signaling pathway [38]. Recent research studies had demonstrated that the activation of PKB causes the activation of mTORC1 and its downstream protein p70S6K, which are critical regulators of cell growth, cell proliferation, and protein synthesis [39, 40].

Consequently, the management of diabetes mellitus with AqTh and EaTh compounds could have helpful effects on renal function in diabetic patients.

Evidence suggests that in diabetic rats, a complex alteration in the activities of antioxidant enzymes was observed [41]. This alteration leads to tissue damage and plays an important role in the pathogenesis of diabetic complications. In the present study, liver histopathological results show that untreated diabetic rats develop liver steatosis characterized by lipid droplets. Other studies found that the liver was necrotized in STZ-induced diabetic rats [42]. STZ is considered a classic model of diabetes induction because it provokes toxicity to pancreatic *β*-cells and cellular death [43], resulting in hypoinsulinemia and hyperglycemia. Insulin plays important metabolic role as a suppressor of lipolysis in adipose tissue [44]. Thus, hypoinsulinemia in diabetes leads to an increased release of free fatty acids (FFAs) in the bloodstream [45] and influx of acids to the liver. Intrahepatic triglyceride accumulation occurs when the influx of lipids to the liver surpasses the hepatic capacity to export triglycerides to the bloodstream [45] which causes the development of hepatic steatosis as diabetic complication [46].

In the present study, the histopathological data show that the medium-term oral administration of 200 mg/kg AqTh or 50 mg/kg EaTh protects significantly the liver under the steatosis via decreasing lipid droplet accumulation. Similar findings were reported by Eliza et al. [47] where *Costus speciosus* decreased hepatic disorder. In addition, other studies showed that *Camellia oleifera* seed ameliorates hepatic steatosis via the regulation of FFA [48] and curcumin reduces steatohepatitis and lipid deposition through being an antioxidant and exerting anti-inflammatory and free radical scavenger roles [49]. However, acarbose treatment did not have this liver protective propriety. This confirms the side effect of this drug in the liver [50].

Furthermore, *T. hirsuta* contains sterols, coumarins, terpenes, tannins, aliphatic alcohol, lactone, alkanes, and alkanols [51]. Interestingly, AqTh and EaTh antihyperglycemic and antidiabetic effects might be due to the presence of these phytochemicals or other unknown compounds.

## 5. Conclusions

In conclusion, our study has shown that the medium-term administration of AqTh and EaTh of *T. hirsuta* had the potential to decrease blood glucose in STZ-induced diabetic rats. The antihyperglycemic effect of EaTh may be partially explained by the inhibition of intestinal *α*-glucosidase activity. And the AqTh antihyperglycemic activity can be attributed to the stimulation of insulin secretion from pancreatic islet cells. In addition, AqTh and EaTh have shown a crucial preventive effect against renal fibrosis and liver steatosis complications. Thus, these findings suggest that AqTh and EaTh could be considered as an efficient oral antidiabetic treatment. Currently, our studies are focused on the isolation and identification of the active compounds of EaTh and AqTh, which are responsible for the antidiabetic effect of *T. hirsuta*.

## Figures and Tables

**Table 1:** Effect of 200 mg/kg AqTh, 50 mg/kg EaTh, and 10 mg/kg acarbose on daily water and food intake and on body weight gain, in STZ-induced diabetic rats after 4-week treatment.

Groups	Water intake (ml/24 h)		Food intake (g/24 h)		Body weight gain (g)
	Before	After	Before	After	
Normal	24.33 ± 4.34	33.00 ± 3.53^*∗*^	18.33 ± 3.00	21.90 ± 1.41	22.88 ± 6.74
Diabetic	70.20 ± 13.64	69.20 ± 14.25	22.14 ± 4.87	29.50 ± 4.96^*∗*^	−6.04 ± 11.10^#^
D + acarbose	56.80 ± 13.08	64.40 ± 15.26	17.30 ± 2.92	28.78 ± 4.20^*∗∗*^	11.82 ± 4.82
D + AqTh	52.40 ± 6.20	56.60 ± 4.97	21.04 ± 2.89	29.11 ± 1.82^*∗*^	21.88 ± 2.39^$^
D + EaTh	50.40 ± 13.00	80.60 ± 14.42	19.38 ± 4.40	34.00 ± 3.23^*∗*^	14.14 ± 6.05

Each value is mean ± SEM. *n* = 5-6 rats. AqTh: aqueous extract; EaTh: ethyl acetate fraction. ^#^*p* < 0.05 compared with the normal group. ^$^*p* < 0.05 compared with the diabetic group.^*∗*^*p* < 0.05 and ^*∗∗*^*p* < 0.01 compared with before treatment in the same group. All statistical tests were used with one-tail *p* value.

**Table 2:** Effect of 200 mg/kg AqTh, 50 mg/kg EaTh, and 10 mg/kg acarbose on urinary parameters in STZ-induced diabetic rats after 4 weeks treatment.

Groups	Urinary volume (ml/24 h)		Glycosuria (g/l)	Urinary creatinine (mg/dl/24 h)
	Before	After		
Normal	10.83 ± 0.84	10.12 ± 0.93^§^	0	41.00 ± 3.42
Diabetic	49.20 ± 12.30	52.20 ± 21.12^§^	36.82 ± 7.75^*∗∗∗*^	61.60 ± 8.63^*∗*^
D + acarbose	36.40 ± 12.71	36.84 ± 12.06^§^	29.09 ± 11.90	52.00 ± 8.94
D + AqTh	16.40 ± 4.16	27.72 ± 6.47^§^	17.61 ± 10.24	31.20 ± 9.75^#^
D + EaTh	24.80 ± 7.26	35.40 ± 11.82^§^	19.11 ± 11.81	47.20 ± 10.46

Each value is mean ± SEM. *n* = 5-6 rats. AqTh: aqueous extract; EaTh: ethyl acetate fraction. ^§^*p* > 0.05 compared with before treatment in the same group. ^*∗*^*p* < 0.05 and ^*∗∗∗*^*p* < 0.001 compared with the normal group. ^#^*p* < 0.05 compared with the diabetic group. All statistical tests were used with one-tail *p* value.

**Table 3:** Effect of 200 mg/kg AqTh, 50 mg/kg EaTh, and 10 mg/kg acarbose on liver glycogen, glycosylated hemoglobin, triglyceride, and total cholesterol in STZ-induced diabetic rats after 4-week treatment.

Groups	Liver glycogen (mg/g tissue)	Glycosylated hemoglobin (HbA1c%)	Triglycerides (mg/dl)	Total cholesterol (mg/dl)
Normal	3.97 ± 1.37	4.37 ± 0.24	38.88 ± 7.38	37.48 ± 5.36
Diabetic	0.52 ± 0.12^*∗*^	5.36 ± 0.40^*∗*^	37.48 ± 8.13^ns^	45.76 ± 9.13^ns^
D + acarbose	1.19 ± 0.77^ns/$^	Not measured	57.61 ± 6.10	46.54 ± 11.82
D + AqTh	0.51 ± 0.01^*∗*/$^	4.61 ± 0.18^ns/$^	36.74 ± 4.01	49.97 ± 5.10
D + EaTh	0.65 ± 0.09^*∗*/$^	5.98 ± 0.79^*∗*/$^	34.78 ± 6.62	42.79 ± 6.02

Each value is mean ± SEM. *n* = 4–6 rats. AqTh: aqueous extract; EaTh: ethyl acetate fraction. ^ns^*p* > 0.05 and ^*∗*^*p* < 0.05 compared with the normal group. ^$^*p* > 0.05 compared with the diabetic group. All statistical tests were used with one-tail *p* value.

**Table 4:** Effect of 200 mg/kg AqTh, 50 mg/kg EaTh, and 10 mg/kg acarbose on kidney, liver, and heart weight in STZ-induced diabetic rats after 4-week treatment.

Groups	Kidney (g)	Liver (g)	Heart (g)
Normal	0.34 ± 0.01	3.22 ± 0.09	0.35 ± 0.01
Diabetic	0.44 ± 0.04^*∗*^	3.15 ± 0.13^ns^	0.31 ± 0.02^ns^
D + acarbose	0.46 ± 0.03^*∗∗*/$^	4.18 ± 0.46^*∗*/#^	0.39 ± 0.02^ns/#^
D + AqTh	0.39 ± 0.02^*∗*/$^	3.18 ± 0.30^ns/$^	0.32 ± 0.01^ns/$^
D + EaTh	0.39 ± 0.02^*∗*/$^	3.09 ± 0.14^ns/$^	0.33 ± 0.01^ns/$^

Each value is mean ± SEM. *n* = 5-6 rats. AqTh: aqueous extract; EaTh: ethyl acetate fraction. ^ns^*p* > 0.05, ^*∗*^*p* < 0.05, and ^*∗∗*^*p* < 0.01 compared with the normal group. ^$^*p* > 0.05 and ^#^*p* < 0.05 compared with the diabetic group. All statistical tests were used with one-tail *p* value.

## Data Availability

The data used to support the findings of this study are available on request from the authors.

## Abbreviations

AqTh: Aqueous extract of *Thymelaea hirsuta*
DM1: Type 1 diabetes
DM2: Type 2 diabetes
EaTh: Ethyl acetate fraction of *Thymelaea hirsuta*
ECM: Extracellular matrix
ESRD: End-stage renal disease
FFA: Free fatty acid
mTOR: Mammalian target of rapamycin
PKB: Protein kinase B
STZ: Streptozotocin
*T. hirsuta*: *Thymelaea hirsuta*
UMPO: Herbarium University Mohamed Premier of Oujda, Morocco
WHO: World Health Organization.

## References

[1] Koyuturk M., Ozsoy-Sacan O., Bolkent S., Yanardag R. (2005). Effect of glurenorm on immunohistochemical changes in pancreatic *β*-cells of rats in experimental diabetes. *Indian Journal of Experimental Biology*.

[2] Hadjadj S., Duly-Bouhanick B., Bekherraz A. (2004). Serum triglycerides are a predictive factor for the development and the progression of renal and retinal complications in patients with type 1 diabetes. *Diabetes & Metabolism*.

[3] Verspohl E. J. (2002). Recommended testing in diabetes research. *Planta Medica*.

[4] Colca J. R. (2006). Insulin sensitizers may prevent metabolic inflammation. *Biochemical Pharmacology*.

[5] Bailey C., Day C. (1989). Traditional plant medicines as treatments for diabetes. *Diabetes Care*.

[6] World Health Organization (1994). WHO study group on diabetes mellitus.

[7] Bellakhdar J., Claisse R., Fleurentin J., Younos C. (1991). Repertory of standard herbal drugs in the Moroccan pharmacopoea. *Journal of Ethnopharmacology*.

[8] Ziyyat A., Legssyer A., Mekhfi H., Dassouli A., Serhrouchni M., Benjelloun W. (1997). Phytotherapy of hypertension and diabetes in oriental Morocco. *Journal of Ethnopharmacology*.

[9] Bnouham M., Mekhfi H., Legssyer A., Ziyyat A. (2002). Ethnopharmacology forum: medicinal plants used in the treatment of diabetes in Morocco. *International Journal of Diabetes and Metabolism*.

[10] Eddouks M., Maghrani M., Lemhadri A., Ouahidi M.-L., Jouad H. (2002). Ethnopharmacological survey of medicinal plants used for the treatment of diabetes mellitus, hypertension and cardiac diseases in the south-east region of Morocco (Tafilalet). *Journal of Ethnopharmacology*.

[11] Bnouham M., Merhfour F. Z., Legssyer A., Mekhfi H., Maallem S., Ziyyat A. (2007). Antihyperglycemic activity of *Arbutus unedo*, *Ammoides pusilla* and *Tymelaea hirsuta*. *Pharmazie*.

[12] Bnouham M., Benalla W., Bellahcen S. (2012). Antidiabetic and antihypertensive effect of a polyphenol rich-fraction of Thymeleae hirsuta L. in a model of neonatal streptozotocin-induced diabetic and L-NAME hypertensive rats. *Journal of Diabetes*.

[13] Abid S., Lekchiri A., Mekhfi H. (2014). Inhibition of *α*-glucosidase and glucose intestinal absorption by *Thymelaea hirsuta* fractions. *Journal of Diabetes*.

[14] World Health Organization (WHO) Chronicle, 39, 51–56, 1985

[15] Sancheti S., Sancheti S., Bafna M., Seo S.-Y. (2010). Antihyperglycemic, antihyperlipidemic, and antioxidant effects of *Chaenomeles sinensis* fruit extract in streptozotocin-induced diabetic rats. *European Food Research and Technology*.

[16] Salahuddin M., Jalalpure S. S. (2010). Antidiabetic activity of aqueous fruit extract of *Cucumis trigonus* Roxb. in streptozotocin-induced-diabetic rats. *Journal of Ethnopharmacology*.

[17] Patel M. B., Mishra S. M. (2012). Magnoflorine from *Tinospora cordifolia* stem inhibits *α*-glucosidase and is antiglycemic in rats. *Journal of Functional Foods*.

[18] Ong K. C., Khoo H.-E. (2000). Effects of myricetin on glycemia and glycogen metabolism in diabetic rats. *Life Sciences*.

[19] Mayer P., Mitt Zool Stn, Neapel, 1896, 12, 303

[20] Kiernan J. A. (2008). *Histological and Histochemical Method (Theory and Practice)*.

[21] Goldner J. (1938). A modification of the mass on trichrome technique for routine laboratory purposes. *The American Journal of Pathology*.

[22] Karatug A., Kaptan E., Bolkent S., Mutlu O., Yanardag R. (2013). Alterations in kidney tissue following zinc supplementation to STZ-induced diabetic rats. *Journal of Trace Elements in Medicine and Biology*.

[23] Al-Eryani M. A. Y., Naik P. R. (2007). Antidiabetic activity of stem extracts of *Tinospora cordifolia* on streptozotocin induced diabetic Wistar rat. *Biosci Biotechnol Res Asia*.

[24] Yin D., Tao J., Lee D. D. (2006). Recovery of islet -cell function in streptozotocin- induced diabetic mice: an indirect role for the spleen. *Diabetes*.

[25] Jamshid M., Prakash R. N. (2012). The histopathologic effects of *Morus alba* leaf extract on the pancreas of diabetic rats. *Turkish Journal of Biology*.

[26] Poongunran J., Perera H., Fernando W., Jayasinghe L., Sivakanesan R. (2015). *α*-glucosidase and *α*-amylase inhibitory activities of nine Sri Lankan antidiabetic plants. *British Journal of Pharmaceutical Research*.

[27] Vinodhini V., Himaja M., Saraswathi S. V., Das P. (2015). In vitro antidiabetic activity of *Tragia involucrata* Linn. leaf extracts. *International Journal of Research in Ayurveda & Pharmacy*.

[28] Chelladurai G. R. M., Chinnachamy C. (2018). Alpha amylase and alpha glucosidase inhibitory effects of aqueous stem extract of *Salacia oblonga* and its GC-MS analysis. *Brazilian Journal of Pharmaceutical Sciences*.

[29] Bnouham M., Benalla W., Bellahcen S. (2012). Antidiabetic and antihypertensive effect of a polyphenol-rich fraction of *Thymelaea hirsuta* L. in a model of neonatal streptozotocin-diabetic and NG-nitro-l-arginine methyl ester-hypertensive rats. *Journal of Diabetes*.

[30] Giorda C. B., Carnà P., Salomone M. (2018). Ten-year comparative analysis of incidence, prognosis, and associated factors for dialysis and renal transplantation in type 1 and type 2 diabetes versus non-diabetes. *Acta Diabetologica*.

[31] Epstein M., Sowers J. R. (1992). Diabetes mellitus and hypertension. *Hypertension*.

[32] Vinikor F. (1994). Is diabetes a public health disorder?. *Diabetes Care*.

[33] Mason R. M., Wahab N. A. (2003). Extracellular matrix metabolism in diabetic nephropathy. *Journal of the American Society of Nephrology*.

[34] Ziyadeh F. N. (2004). Mediators of diabetic renal disease: the case for TGF-beta as the major mediator. *Journal of the American Society of Nephrology*.

[35] Schena F. P., Gesualdo L. (2005). Pathogenetic mechanisms of diabetic nephropathy. *Journal of the American Society of Nephrology*.

[36] Baluchnejadmojarad T., Roghani M. (2003). Endothelium-dependent and independent effect of aqueous extract of garlic on vascular reactivity on diabetic rats. *Fitoterapia*.

[37] Gondwe M., Kamadyaapa D. R., Tufts M., Chuturgoon A. A., Musabayane C. T. (2008). Sclerocarya birrea [(A. Rich.) Hochst.] [Anacardiaceae] stem-bark ethanolic extract (SBE) modulates blood glucose, glomerular filtration rate (GFR) and mean arterial blood pressure (MAP) of STZ-induced diabetic rats. *Phytomedicine*.

[38] Lu Q., Zuo W.-Z., Ji X.-J. (2015). Ethanolic *Ginkgo biloba* leaf extract prevents renal fibrosis through Akt/mTOR signaling in diabetic nephropathy. *Phytomedicine*.

[39] Hers I., Vincent E. E., Tavaré J. M. (2011). Akt signalling in health and disease. *Cellular Signalling*.

[40] Ma X. M., Blenis J. (2009). Molecular mechanisms of mTOR-mediated translational control. *Nature Reviews Molecular Cell Biology*.

[41] Scott J. A., King G. L. (2004). Oxidative stress and antioxidant treatment in diabetes. *Annals of the New York Academy of Sciences*.

[42] Ohaeri O. C. (2001). Effect of garlic oil on the levels of various enzymes in the serum and tissue of streptozotocin diabetic rats. *Bioscience Reports*.

[43] Lenzen S. (2007). The mechanisms of alloxan- and streptozotocin-induced diabetes. *Diabetologia*.

[44] Rosa L. R. d. O., Kaga A. K., Barbanera P. O., Queiroz P. M., do Carmo N. O. L., Fernandes A. A. H. (2018). Beneficial effects of *N*-acetylcysteine on hepatic oxidative stress in streptozotocin-induced diabetic rats. *Canadian Journal of Physiology and Pharmacology*.

[45] Adiels M., Taskinen M.-R., Borén J. (2008). Fatty liver, insulin resistance, and dyslipidemia. *Current Diabetes Reports*.

[46] Cusi K., Arun J. S., Shuyu Z. M. S. (2017). Non-alcoholic fatty liver disease (NAFLD) prevalence and its metabolic associations in patients with type 1 diabetes and type 2 diabetes. *Diabetes, Obesity and Metabolism*.

[47] Eliza J., Daisy P., Ignacimuthu S., Duraipandiyan V. (2009). Antidiabetic and antilipidemic effect of eremanthin from Costus speciosus (Koen.)Sm., in STZ-induced diabetic rats. *Chemico-Biological Interactions*.

[48] Yang H.-Y., Yeh W.-J., Ko J., Chen J.-R. (2019). *Camellia oleifera* seed extract attenuated abdominal and hepatic fat accumulation in rats fed a high-fat diet. *Applied Physiology, Nutrition, and Metabolism*.

[49] Hismiogullari S. E., Hismiogullari A. A., Sunay F. B. (2014). The protective effect of curcumin on carbon tetrachloride induced liver damage. *Revue de Médecine Vétérinaire*.

[50] Liu L., Deseo M. A., Morris C., Winter K. M., Leach D. N. (2011). Investigation of *α*-glucosidase inhibitory activity of wheat bran and germ. *Food Chemistry*.

[51] Dohou N., Yamni K., Tahrouch S., Idrissi Hassani L. M., Badoc A., Gmira N. (2003). Screening phytochimique d’une plante endémique ibero-marocaine, *Thymelaea lythroides*. *Bulletin de la Société de Pharmacie de Bordeaux*.

